# Supplementary figures and images for: In silico screening of 393 mutants facilitates enzyme engineering of amidase activity in CalB
Source: PeerJ. 2013 Aug 29;1:e145. doi: 10.7717/peerj.145 (PMC3757469; doi:10.7717/peerj.145)

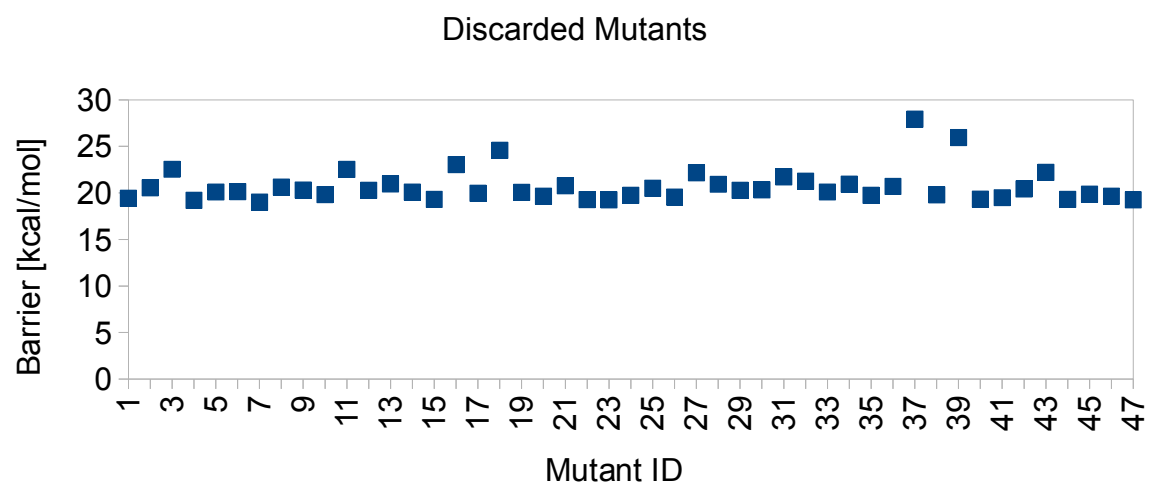

Supplement: Figure S1 — Single mutants: 0; Double mutants: 8; Triple mutants: 20; Four-fold mutants: 19; Total: 47. [file peerj-01-145-s001.pdf]

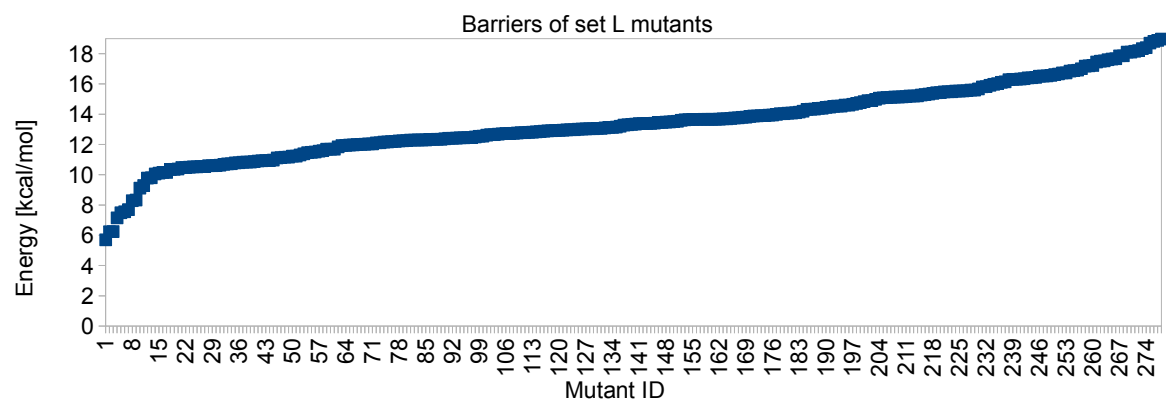

Supplement: Figure S2 — Single: 13; Double mutants: 50; Triple mutants: 101; Four-fold mutants: 114: Total: 278. [file peerj-01-145-s002.pdf]

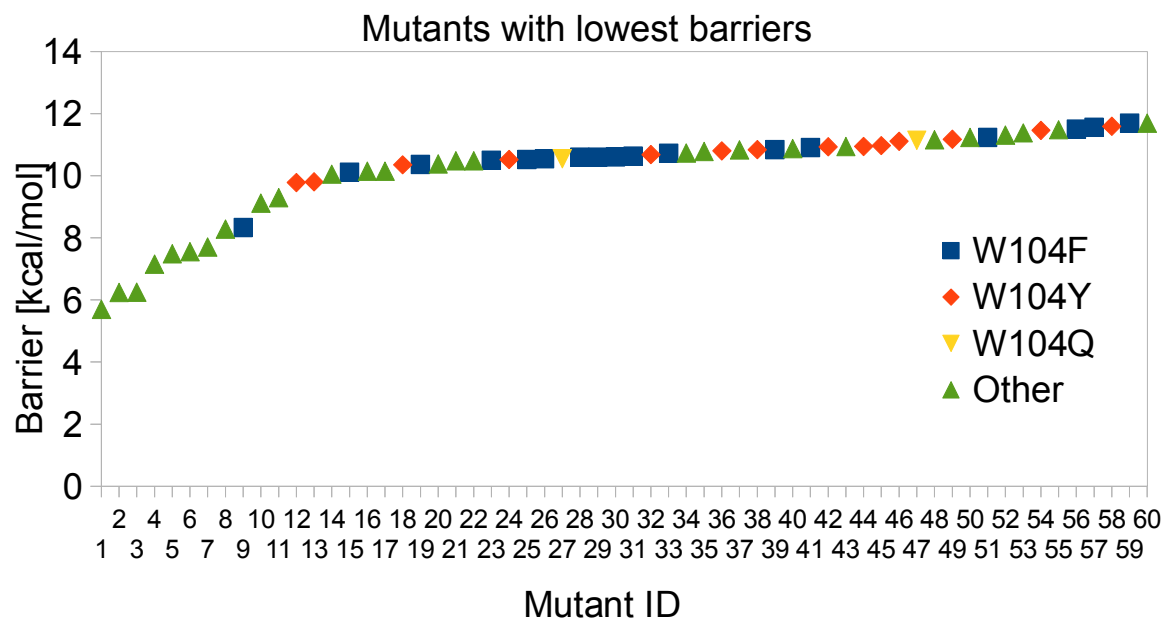

Supplement: Figure S3 — 33 out of 60 contain a mutation of W104 (W104F: 17, W104Y: 14, W104Q: 2). [file peerj-01-145-s003.pdf]
